# Supplementary material for: Smart strategies to navigate turbulent odor plumes reorienting to local wind
Source: ArXiv. 2026 Jul 29:arXiv:2605.21329v2. Originally published 2026 May 20. Preprint. [Version 2] (PMC13229087)
Supplement: Supplementary file 1 [file NIHPP2605.21329v2-supplement-1.pdf]

SUPPLEMENTAL MATERIAL

1. Supplementary figures

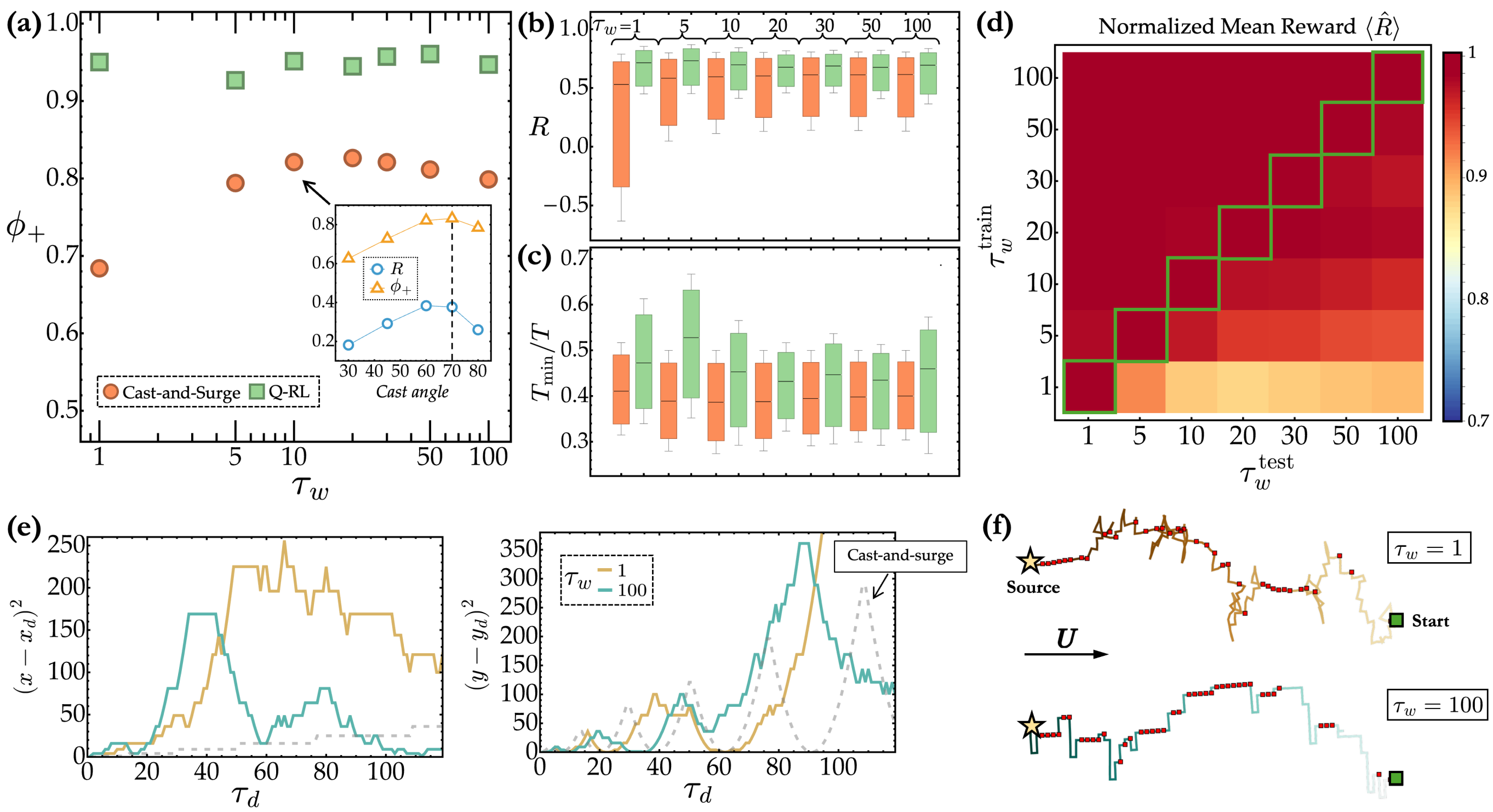

FIG. 6. **Performance of learned policies versus cast-and-surge in the stronger mean wind regime ($U/u_{\rm rms} = 2$). (a)** Success fraction ϕ_+ as a function of wind-estimation memory time τ_w for Q-RL (green squares) and cast-and-surge (red circles). Q-RL consistently outperforms the baseline across all τ_w, with $\phi_+ \gtrsim 0.93$, while cast-and-surge achieves $\phi_+ \approx 0.8$ at its optimally tuned casting angle (inset). The near-flat dependence on τ_w confirms that a strong mean flow renders the learned policy largely insensitive to wind-estimation memory. **(b)** Box plots of the cumulative reward R for Q-RL (green) and cast-and-surge (red). Q-RL systematically achieves higher, less variable rewards across all τ_w. **(c)** Box plots of $T_{\rm min}/T$ conditioned on successful trajectories. Arrival-time distributions of Q-RL and cast-and-surge are broadly comparable, indicating that Q-RL's advantage lies primarily in its higher success rate rather than faster navigation. **(d)** Cross-testing matrix of the normalized mean reward $\langle \hat{R} \rangle$ for policies trained at $\tau_w^{\rm train}$ (rows) and deployed at $\tau_w^{\rm test}$ (columns), without retraining. Rewards are normalized by the matched diagonal values (green outline), i.e., the performance of each policy when tested with the same memory used during training. Except for $\tau_w^{\rm train} = 1$, the matrix is close to unity off-diagonal, demonstrating broad transfer robustness; the strong mean flow makes τ_w a secondary factor for all but the shortest memory. **(e)** Conditional mean-squared displacements with respect to the last detection position (x_d, y_d): longitudinal $(x - x_d)^2$ (left) and transverse $(y - y_d)^2$ (right) as functions of τ_d, for $\tau_w = 1$ (gold), $\tau_w = 100$ (teal), and cast-and-surge (dashed grey). Both memory extremes show similar profiles, with dominant transverse casting and infrequent longitudinal reversals, in contrast to the mild-wind regime where the two extremes differ markedly. **(f)** Representative trajectories for $\tau_w = 1$ (top, gold) and $\tau_w = 100$ (bottom, teal) from the same initial position (green square); source marked by a star, mean wind by $\boldsymbol{U}$, odor hits by red squares. Both agents perform casting with infrequent wind-direction reversals, consistent with panel (e), confirming that, in the strong mean wind regime, the learned policy does not strongly adapt to wind-estimation memory.

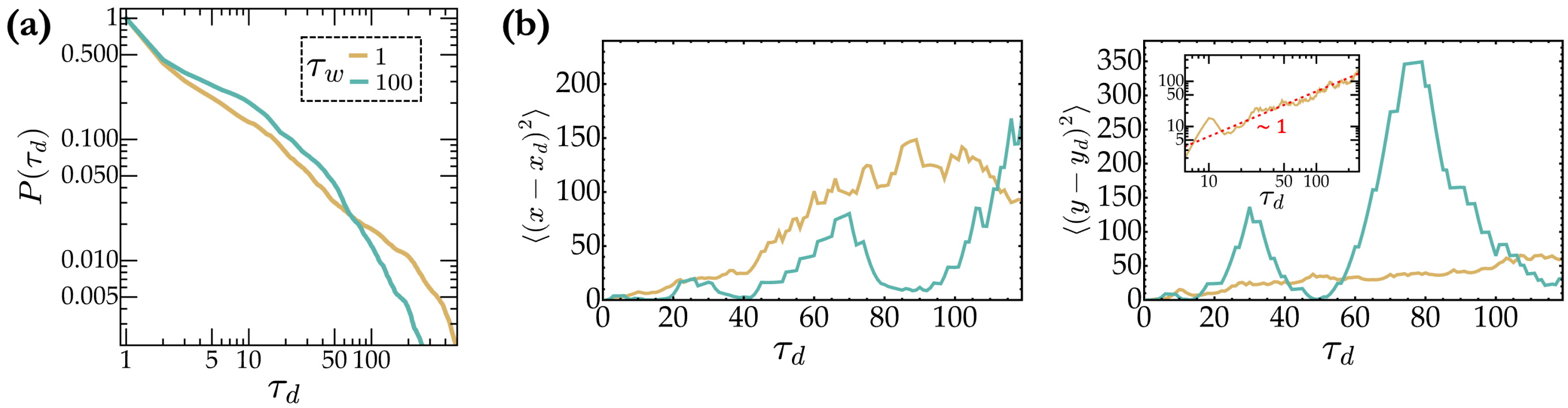

FIG. 7. **Mean-squared-displacement analysis of learned strategies in the mild mean wind regime ($U/u_{\rm rms} = 1$).** Results are shown for $\tau_w = 1$ (gold) and $\tau_w = 100$ (teal). **(a)** State-visitation probability $P(\tau_d)$ normalized at its value at $\tau_d = 0$ as a function of the time τ_d elapsed since the last odor detection, on a log-log scale. For both memory times, $P(\tau_d)$ decays rapidly and falls below ~ 1–2% for $\tau_d \gtrsim 100$, confirming that long detection-free intervals are rarely visited; the displacement analysis in panels (b)–(c) is therefore restricted to this functionally active range. **(b)** Conditional mean-squared displacements in the allocentric reference frame averaged over different flow realizations and initial conditions. Top: longitudinal component $\langle(x - x_d)^2\rangle$. Bottom: transverse component $\langle(y - y_d)^2\rangle$. For $\tau_w = 100$, the motion is strongly anisotropic at large τ_d, with enhanced transverse excursions reflecting active casting and longitudinal oscillations reflecting periodic backtracking. For $\tau_w = 1$, the transverse component grows approximately linearly with τ_d (inset, log-log scale; dashed red line indicates diffusive scaling $\sim \tau_d^1$). This diffusive behavior admits a transparent analytical interpretation. When $\tau_w = 1$, the wind estimate $\bar{\boldsymbol{U}}$ tracks instantaneous turbulent fluctuations and decorrelates rapidly between successive steps. Each action is therefore rotated by a nearly independent random angle, so that the crosswind component of consecutive displacements is uncorrelated: the transverse displacement accumulates as a sum of independent random increments, yielding diffusive growth $\langle(y - y_d)^2\rangle \sim \tau_d$ by the central limit theorem. The short-memory agent has thus learned to exploit the fast angular decorrelation of the flow to execute a random walk in the crosswind direction, achieving lateral coverage through fluctuation-driven diffusion rather than directed casting.

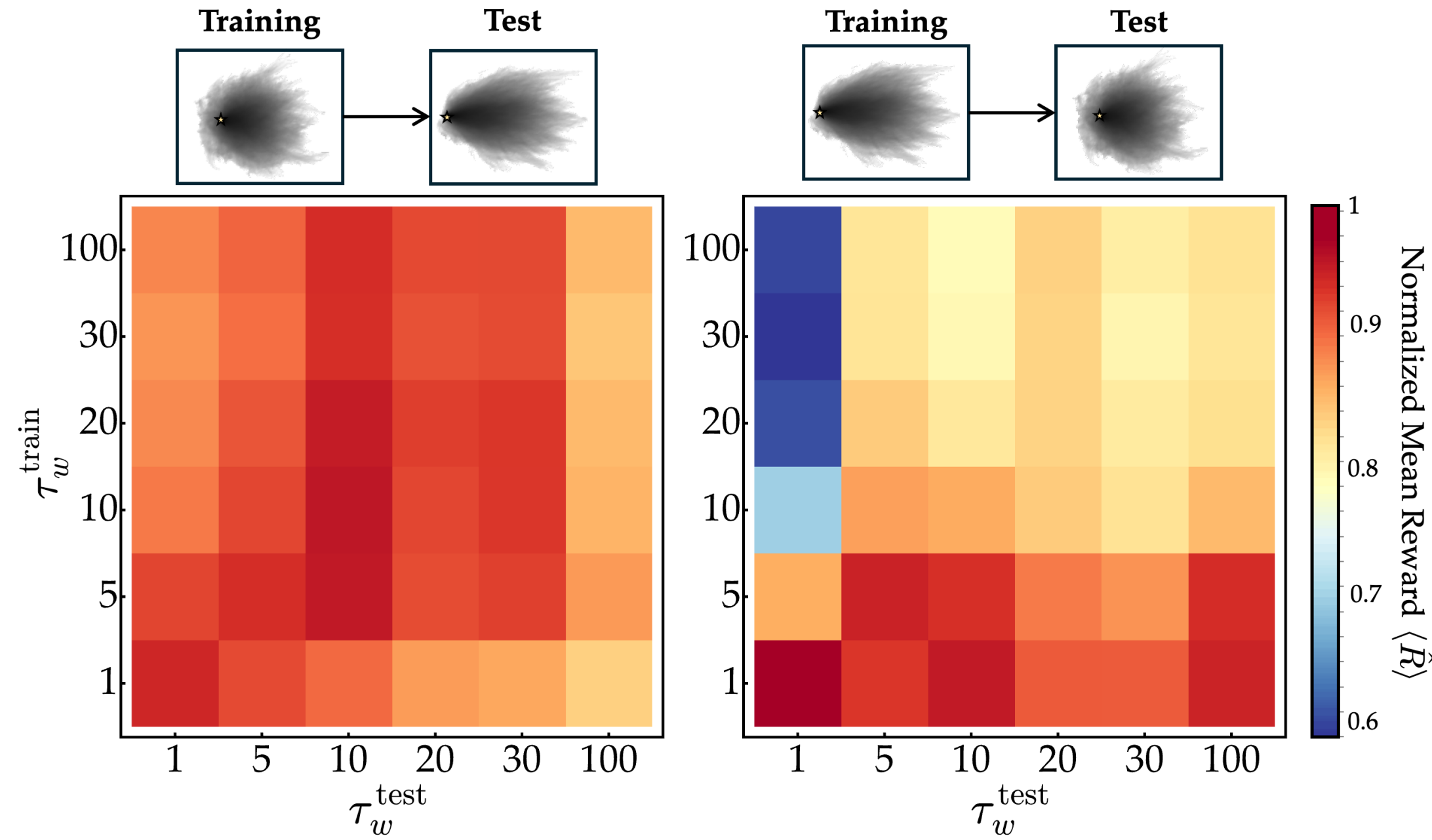

FIG. 8. **Cross-environment transfer of learned policies between the mild ($U/u_{\text{rms}} = 1$) and strong ($U/u_{\text{rms}} = 2$) mean wind regimes.** Each matrix shows the normalized mean reward $\langle \hat{R} \rangle$ when a policy trained in one flow environment is deployed in a different one, as a function of the wind-estimation memory time used during training (τ_w^{train}, rows) and during testing (τ_w^{test}, columns). Each entry is normalized by the performance achieved when the same policy is tested in the environment it was trained on, so that values below unity directly quantify the performance loss due to environment mismatch. The schematics above each matrix indicate the direction of transfer: the training plume (left thumbnail) is transported to the test plume (right thumbnail). **(Left)** Transfer from the mild-wind environment ($U/u_{\text{rms}} = 1$, training) to the strong-wind environment ($U/u_{\text{rms}} = 2$, test). The matrix is uniformly warm-coloured and close to unity across all $(\tau_w^{\text{train}}, \tau_w^{\text{test}})$ pairs, demonstrating that policies learned in the harder, mild-wind regime transfer essentially without loss to the easier, strong-wind regime. This asymmetry is physically natural: a policy trained under a weaker mean flow, where wind-direction estimation is a genuine challenge and turbulent fluctuations are comparably strong, develops a robust search strategy that remains effective when a more reliable directional cue becomes available. **(Right)** Transfer from the strong-wind environment ($U/u_{\text{rms}} = 2$, training) to the mild-wind environment ($U/u_{\text{rms}} = 1$, test). Performance degrades substantially for policies trained at large $\tau_w^{\text{train}} \geq 10$. Policies trained at short memory ($\tau_w^{\text{train}} \leq 5$) transfer more successfully, recovering near-diagonal performance at small τ_w^{test}. This degradation reflects the fact that the strong-wind environment is an easier task: strategies learned there over-exploit the reliable mean-flow direction and rely on long-memory wind estimates to navigate, developing a policy that is too rigid to cope with the stronger directional uncertainty and larger turbulent fluctuations encountered in the mild-wind regime. Together, the two panels establish a clear asymmetry in cross-environment generalization: for practical deployment of olfactory navigation agents across variable flow conditions, training in the more demanding, lower wind-to-turbulence-ratio regime is the preferable strategy.

Appendix D: Details on the Q-learning algorithm

Algorithm 1 Q-learning for olfactory search: the input comprises concentration $c(\boldsymbol{x}, t)$ and velocity fields $\boldsymbol{v}(\boldsymbol{x}, t)$ from DNS data, source position $\boldsymbol{x}_s$ and radius r_s, episode time horizon T_H, discount factor γ, wind-estimation memory time τ_w, number of training episodes N_{train}, initial and final learning and exploration rates $\alpha_0, \alpha_f, \varepsilon_0, \varepsilon_f$.

Initialize
$Q(s,a) \leftarrow -1 \quad \forall\, s \in \{0,\ldots,T_H-1\},\ a \in \mathcal{A} = \{\boldsymbol{e}_x, -\boldsymbol{e}_x, \boldsymbol{e}_y, -\boldsymbol{e}_y\}$ ▷ Initialize Q-table

$B = 256, \quad \alpha_k = \alpha_0(\alpha_f/\alpha_0)^{k/N_{\text{train}}}, \quad \varepsilon_k = \varepsilon_0(\varepsilon_f/\varepsilon_0)^{k/N_{\text{train}}}$ ▷ Agents running in parallel per episode and decaying schedules

for each episode $k = 0, 1, \ldots, N_{\text{train}}$ **do**
  Draw random time index t_0 uniformly from valid range
  for each agent $i = 1, \ldots, B$ **in parallel do**
    $\boldsymbol{x}^{(i)}(0) \leftarrow$ SampleInitialCondition$(c,\, c_{\text{thr}}, t_0)$
    $s^{(i)} \leftarrow$ UpdateState$(c(\boldsymbol{x}^{(i)}(0),\, t_0))$ ▷ Encode initial observation into discrete state
    $\boldsymbol{\mu}^{(i)} \leftarrow$ InitFlowMemory$(\boldsymbol{v},\, \boldsymbol{x}^{(i)}(0),\, t_0,\, \tau_w)$ ▷ Initialize exponential moving average (EMA) of flow velocity
  end for
  for each time step $t = 0, 1, \ldots, T_H - 1$ **do**
    for each active agent $i = 1, \ldots, B$ **in parallel do**
      $a^{(i)} \leftarrow$ SelectAction$(Q,\, s^{(i)},\, \varepsilon_k)$ ▷ ε-greedy action selection
      $\boldsymbol{\mu}^{(i)} \leftarrow (1-\alpha_M)\,\boldsymbol{\mu}^{(i)} + \alpha_M\, \boldsymbol{v}(\boldsymbol{x}^{(i)}(t), t_0^{(i)} + t), \quad \alpha_M = 1 - e^{-1/\tau_w}$ ▷ Update flow EMA
      $\boldsymbol{x}^{(i)}(t+1) \leftarrow \boldsymbol{x}^{(i)}(t) + R\Big(\text{atan2}(\mu_y^{(i)}, \mu_x^{(i)})\Big)\, a^{(i)}$ ▷ Rotate action along mean flow and move
      if $|\boldsymbol{x}^{(i)}(t+1) - \boldsymbol{x}_s|_1 \le r_s$ **then**
        $r^{(i)} \leftarrow 1, \quad s^{(i)\prime} \leftarrow -1$ ▷ Terminal reward and state upon source found
      else
        $r^{(i)} \leftarrow \gamma - 1$ ▷ Immediate reward
        $s^{(i)\prime} \leftarrow$ UpdateState$(c\Big(\boldsymbol{x}^{(i)}(t+1),\, t_0^{(i)} + t + 1\Big))$ ▷ Sample concentration and update state
      end if
    end for
    UpdateQ$(Q,\, \{s^{(i)}\},\, \{a^{(i)}\},\, \{s^{(i)\prime}\},\, \{r^{(i)}\},\, \alpha_k,\, \gamma)$ ▷ Single Q-table updated with all B transitions
    $s^{(i)} \leftarrow s^{(i)\prime} \quad \forall\, i$ with $s^{(i)\prime} \ne -1$
    if $s^{(i)\prime} = -1\ \forall i$ **then break**
    end if ▷ All agents have reached the source
  end for
end for

function SampleInitialCondition$(c,\, c_{\text{thr}}, t_0)$
  $\mathcal{X}_0 \leftarrow \{\boldsymbol{x} : c(\boldsymbol{x}, t_0) > c_{\text{thr}}\} \setminus \mathcal{B}(\boldsymbol{x}_s, r_s)$ ▷ Valid positions above threshold, away from source
  Draw $\boldsymbol{x}(0)$ uniformly from $\mathcal{X}_0$
  return $\boldsymbol{x}(0)$, t_0
end function

function InitFlowMemory$(\boldsymbol{v},\, \boldsymbol{x},\, t_0,\, \tau_w)$
  $\boldsymbol{\mu} \leftarrow \boldsymbol{v}(\boldsymbol{x},\, t_0 - \tau_w)$
  for $\tau = t_0 - \tau_w + 1, \ldots, t_0$ **do**
    $\boldsymbol{\mu} \leftarrow (1-\alpha_M)\,\boldsymbol{\mu} + \alpha_M\, \boldsymbol{v}(\boldsymbol{x},\, \tau)$ ▷ Warm-up EMA over past M samples
  end for
  return $\boldsymbol{\mu}$
end function

function UpdateState(h)
  return $s \leftarrow \begin{cases} 0 & h \ge c_{\text{thr}} \\ s+1 & \text{otherwise} \end{cases}$ ▷ Odor detection / no odor
end function

function SelectAction$(Q,\, s,\, \varepsilon)$
  return $a \leftarrow \begin{cases} \sim \text{Uniform}(\mathcal{A}) & u < \varepsilon, \quad u \sim \text{Uniform}(0,1) \\ \arg\max_{a'} Q(s, a') & \text{otherwise} \end{cases}$ ▷ Explore: random action / exploit: greedy action
end function

function UpdateQ$(Q,\, \{s^{(i)}\},\, \{a^{(i)}\},\, \{s^{(i)\prime}\},\, \{r^{(i)}\},\, \alpha,\, \gamma)$
  $Q(s^{(i)}, a^{(i)}) \leftarrow (1-\alpha)\, Q(s^{(i)}, a^{(i)}) + \alpha\big[r^{(i)} + \gamma\, \Theta(s^{(i)\prime}) \max_{a'} Q(s^{(i)\prime}, a')\big] \quad \forall\, i : s^{(i)} \ge 0$ ▷ Tabular Q-learning update
end function

Appendix E: Captions of Supplementary Movies

Supplementary Movie S1 | Representative trajectory of the Q-RL agent in the mild mean wind regime ($U/u_{\rm rms} = 1$, $\tau_w = 1$). The agent (gold trajectory) starts at the red square and navigates toward the odor source (green circle), whose emitted scalar concentration field $c(\boldsymbol{x}, t)$ is rendered in the background using a grey-shaded logarithmic color scale. Color intensity along the trajectory encodes elapsed time since departure, with darker gold tones indicating later decision steps. Small red squares mark individual detection events ($\tau_d = 0$), i.e. moments at which the agent registers a non-zero odor signal above threshold. Time is reported above the panel in units of the agent's decision time step. This trajectory corresponds to the episode shown in Fig. 3(c) of the main text.

Supplementary Movie S2 | Representative trajectory of the Q-RL agent in the mild mean wind regime ($U/u_{\rm rms} = 1$, $\tau_w = 100$). Same as *Supplementary Movie S1*, but for an agent operating at $\tau_w = 100$ (teal trajectory).

Supplementary Movie S3 | Representative trajectory of the cast-and-surge agent in the mild mean wind regime ($U/u_{\rm rms} = 1$, $\tau_w = 30$). Same as Supplementary Movie S1, but for the cast-and-surge heuristic with optimal casting angle ($\theta_c = 70°$) and wind-estimate memory time $\tau_w = 30$ (blue trajectory), run on the same episode; the agent fails to locate the source within the time horizon.

Supplementary Movie S4 | Representative trajectory of the Q-RL agent in the isotropic regime ($U/u_{\rm rms} = 0$, $\tau_w = 5$). The agent (yellow-red trajectory) starts at the red square and navigates toward the odor source (green circle), whose emitted scalar concentration field $c(\boldsymbol{x}, t)$ is rendered in the background using a grey-shaded logarithmic color scale. Color intensity along the trajectory encodes elapsed time since departure, with darker red tones indicating later decision steps. Small red squares mark individual detection events. Time is reported above the panel in units of the agent's decision time step. This trajectory corresponds to the episode shown in Fig. 4(d) of the main text.

Supplementary Movie S5 | Representative trajectory of the Q-RL agent in the isotropic regime ($U/u_{\rm rms} = 0$, $\tau_w = 30$). Same as *Supplementary Movie S4*, but for an agent operating at $\tau_w = 30$ (yellow-green trajectory).

Supplementary Movie S6 | Representative trajectory of a spiraling agent in the isotropic regime ($U/u_{\rm rms} = 0$). Same as Supplementary Movie S4, but for the spiral-search heuristic (blue trajectory) with optimal growth rate ($\delta\ell = 8$).

Supplementary Movie S7 | Time evolution of the passive scalar field in the mild mean wind regime ($U/u_{\rm rms} = 1$). The scalar concentration field $c(\boldsymbol{x}, t)$ is shown in log-scale at the full DNS resolution (1024^2 grid), with the source located at the yellow star; the mean wind blows from right to left. The plume is continuously emitted from the point-like source and stretched and folded by the underlying turbulent velocity field into an irregular, intermittent filamentary structure, illustrating the sparse and unpredictable nature of the odor signal encountered by navigating agents. The absorbing penalization mask applied near the domain boundaries, which prevents scalar re-entrance through the periodic boundaries, is visible as the sharp drop in concentration at the edges. Time t is indicated in simulation units above the panel.

Supplementary Movie S8 | Time evolution of the passive scalar field in the isotropic regime ($U/u_{\rm rms} = 0$). Same as Supplementary Movie S7, but in the absence of a mean wind.
